# The role of traditional leadership in disaster management and disaster risk governance: A case of Ugu District Municipality by-laws

**DOI:** 10.4102/jamba.v11i1.802

**Published:** 2019-10-14

**Authors:** Nonhlanhla A. Zamisa, Sybert Mutereko

**Affiliations:** 1 KwaZulu-Natal Department of Cooperative Governance and Traditional Affairs, Durban, South Africa; 2 Discipline of Public Governance, College of Law and Management Studies, University of KwaZulu-Natal, Durban, South Africa

**Keywords:** disaster management, traditional leadership, by-laws

## Abstract

Section 151(2) of the Constitution empowers municipalities in South Africa to pass disaster management-related by-laws. Such by-laws should be specific on the role of traditional leaders, owing to their authority and proximity to the people coupled with their constitutional mandate to preserve customs and traditions. However, their role is often not maximised because of vague and inadequate policies. There has been little or no scholarly attention to the role of traditional leadership and the policy and legal framework that guide their participation in disaster risk management. Employing a comprehensive content analysis of Ugu District Municipality Disaster Management By-law, this article assesses the adequacy of these by-laws on disaster risk governance in the context of collaboration disaster risk reduction. While the Ugu District Municipality Disaster Management By-law provides for the participation of traditional leadership, this study reveals that it is fraught with ambiguities and seemingly vague clauses. For instance, although in Article 5.1.1 the word ‘authorities’ is used, it is not clear whether this refers to traditional leadership or other entities at the local level. In addition, the composition of the Disaster Management Advisory Forum in Ugu does not explicitly include AmaKhosi. While these results add to the rapidly expanding field of disaster risk management, they also suggest several courses of action for policymakers at local government. Such actions might include, but not limited to, a review of the by-laws to address the lack of collaborative essence relative to traditional leaders for optimal disaster risk reduction initiatives targeting traditional communities.

## Introduction

Rural communities are the most vulnerable to disaster risks because of their inability to cope owing to their weak socio-economic conditions (Twigg 2015:3). Traditional leaders have a crucial role to play owing to their authority and proximity to the people and their primary mandate to preserve customs and traditions derived from the Constitution. Moreover, a collaboration between municipalities and traditional institutions is generally an expectation that interfaces with the disaster risk governance actions of the Sendai Framework for Disaster Risk Reduction (SFDRR). Despite the expectation for collaboration between municipalities and traditional leaders, which the SFDRR posits as essential to optimise disaster risk reduction initiatives in rural communities, their recognition often lacks and, moreover, their contribution is not maximised because of vague and inadequate policies (Knoetze 2014:167).

While traditional leaders are supposed to be recognised as a gateway to their communities in line with the entry point as Twigg (2015:125–126) theorises, and playing a central role in the preservation of customs and traditions that can contribute to disaster risk reduction, their role is often understated and overlooked (Knoetze 2014:167). This situation challenges the legitimacy of the global frameworks to which various countries, including South Africa, ascribe. The democratic constitutionality of South Africa that passed a range of statutes contemplating collaborative governance is put to test in circumstances where government conduct deviates. Communities are compromised as they do not enjoy the benefit of being nurtured and guided under the traditional leadership institution, which is part of the public administration and funded largely from the national purse (Selepe 2009:83). This is despite the capacity challenges in municipalities revealed in Wentink and Van Niekerk (2017:8), while the capacity presented by the institution of traditional leadership could well augment this incapacity. Communities, as a result, are exposed to the devastating effects of repeated disaster incidences, which find them vulnerable in the same manner as they were before. These circumstances prevailed in the case for the flood disasters that struck Ugu district in 1987, 2008 and 2011 (Carstens 2011:18–19). No social capital and/or institutional memory prevailed to minimise the flood disaster impact. Community resilience measures could not be traced, and coping mechanisms had not improved from one incidence to the next.

Data from various studies reveal that disasters are mostly induced from past human interaction with the ecosystem, which has a contribution to climate change. Twigg (2015:2), for instance, argues that disaster incidences triggered by natural hazards are mistaken as natural disasters, yet there is no such thing as a natural disaster. According to Deegan (2006:3), ‘humans create the conditions that exacerbate natural disasters’. He further opines that there is a need to forge cooperation and have those affected participating in initiatives aimed at reducing disasters. These studies are in fact consistent with the approach put forward in the global frameworks purporting disaster risk governance measures.

While these studies confirm the need for a multi-sectoral collaborative approach towards disaster risk reduction as contextualised in global frameworks together with the enabling legislative environment prevailing in South Africa, none of them traces the alignment of the local level regulatory and policy prescripts with the accepted global frameworks. This is viewed as a serious omission given that the actions of the public administration are guided through legislative and policy prescripts. In this regard, the collaborative approach towards disaster reduction can only take effect if clearly articulated. In addition, most existing literature on disaster management in South Africa is limited to local surveys that sought the opinions of respondents without a thorough examination of the relevant policies, which ideally should influence action.

To contribute some useful insights into the legislative environment of participatory disaster management in South Africa, this article draws on Ugu District Municipality Disaster Management By-law to understand the stated roles of traditional leadership in disaster risk governance. Following this introduction, the second section presents a brief overview of disaster management legislative framework to provide the policy context of the Ugu District Municipality Disaster Management By-law. The third section considers the theoretical literature on disaster risk management. This includes the outline of the human element in disaster risks, the effects of underdevelopment, commonly associated with rural areas where traditional leadership plays a significant role, on vulnerability to disaster risks and end with an outline of traditional institutions as a reinforcement of disaster risk reduction capacity.

### Disaster management and traditional leadership legislative and framework

The Constitution of the Republic of South Africa recognises the institution of traditional leadership. Section 211(1) of the Constitution states that the role, status and the institution of traditional leadership are recognised. According to Section 212(1), ‘the national legislation may provide for a role for traditional leadership as an institution at local level on matters affecting local communities’. Such matters could include disaster management and mitigation. Although the Constitution provides for the participation of traditional leadership in matters that affect their communities, the operationalisation of such at local level, particularly in by-laws, is not clear. Given this constitutional provision, the explicit role of traditional leadership in disaster management should find a clear and unambiguous expression in local by-laws such as those of Ugu District Municipality.

However, the constitutional provisions for the participation of traditional leadership in matters that affect their communities find expression in the *Municipal Structures Act of 1998*. For instance, Section 81(1) to (5) outlines how traditional leaders are identified for participation in the proceedings of a municipal council. However, the proportion of traditional leadership should not exceed 20% of the number of councillors. Section 81(3) clearly states that (3) when dealing with matters directly affecting an area under traditional leadership, the municipality must give the leader an opportunity to express his or her views on the matter. While this is clear, it is generic and falls short of explaining the actual role of such leadership in disaster management. The *Governance Framework Amendment Act* (No. 41, 2003 Section 20 [1] [l]) is clear in that regard as it states that the national government or the provincial government, through legislative or other mechanisms, should provide a role for traditional councils or traditional leaders in respect of disaster management. While this is clear, such a role is not clearly spelt out in the Ugu District Municipality by-laws.

The governance dimension towards disaster risk reduction in South Africa can be traced back to 1994. The government in this year commenced with the disaster management legislative processes, which resulted in the enactment of the *Disaster Management Act* (Act No. 57 of 2002) (DMA). The *Constitution* bestows an obligation on the *State* to *‘respect, protect, promote and fulfil’* the people’s constitutional right to a safe and healthy environment. Through the legislative authority vested in municipal councils in terms of *Section 151(2) of the Constitution*, municipalities are empowered to pass disaster management-related by-laws. Global frameworks like the *Hyogo Framework for Action* (*HFA*) of 2005 and the Sendai Framework for Disaster Risk Reduction (SFDRR) of 2015 encapsulate disaster risk governance as a multi-sectoral collaborative process. Priority 2 of the SFDRR on *Strengthening Disaster Risk Governance* envisages a shift from the government to the collaborative governance approach. Among required actions is the review of legislative frameworks, regulations and policy directives so as to encapsulate the involvement of public and private sector institutions including indigenous people in addressing disaster risks.

Municipalities, by design, having regard to Section 152(1)(e) of the Constitution are compelled to be participatory (Republic of South Africa 1996). Community systems in South Africa, however, differ given that other parts of the country are formed under the indigenous governance system. History affirms that the institution of traditional leadership is the oldest system and the only form of governance in rural areas, which existed before the colonial invasion (Selepe 2009:103). This governance system, as Selepe (2009:103) points out, stemmed from a social organisation and it was defined by family and kinship trust. A paradigm shift took place during the 19th century when the European colonial masters changed the system in line with their interests and introduced statutes to regulate the institution (Selepe 2009:109). In this regard, the *South African Act of 1909* bestowed authority to the *Governor General* as the ‘Supreme Chief’ and empowered him to establish and divide tribes, and to use his judgement to appoint and overthrow traditional leaders. The *National Party* government subsequent to its rise to power in 1948 entrenched the practice of regulating traditional leaders along with its desires and introduced several pieces of legislative prescripts. Most of these legislative prescripts favoured the interest of the apartheid government rather than protecting the stature and dignity of traditional leaders as leaders in their communities. For instance, the *Bantu Authorities Act* (Act No. 68 of 1951), which was adopted by the apartheid regime for purposes of ‘separate development’, introduced the establishment of ‘tribal authorities’ as a governance system that allowed government to control communities from afar (Fuo 2014:75; De Kadt & Larreguyz 2014:4). The existence of the tribal authorities changed from their initial traditional system and government made it be part of the system of government through statutes. However, both the colonial and apartheid regimes eroded the trust relationship between traditional leaders and their people through the force that they used to entrench the desires of the regimes. People felt the pressure and revolted against their traditional leaders, and this situation intensified during the 1980s through the resistance to the entire Bantustan system with the African National Congress and the United Democratic Front staged throughout the country (De Kadt & Larreguyz 2014:8).

By the time the Multi-Party Negotiating Forum (MNF) was established to negotiate the transition from the apartheid system to a democratic system, traditional leaders had managed to organise themselves into Congress of Traditional Leaders in South Africa (CONTRALESA) that was founded in 1987 as their machinery to resist the Bantustan system as well (De Kadt & Larreguyz 2014:7). Congress of Traditional Leaders in South Africa participated firstly indirectly, based on the relationship established with ANC leaders during the Convention for a Democratic South Africa (CODESA), and later directly through membership on the MNF in 1993 (De Kadt & Larreguyz 2014:8). Their involvement in the transition phase allowed them to advance their position as an institution that resulted in the inclusion of their role in the *Interim Constitution* and ultimately the incorporation of Chapter 12 of the Constitution that provides for the recognition of traditional leaders in the current democratic dispensation (Selepe 2009:117). This recognition compelled legislators to incorporate traditional leaders into the secondary legislative prescripts regulating local governance. In this regard, the collaborative approach is contextualised in relation to the White Paper on Local Government, 1998 as the ‘cooperative model for rural governance’. This model posits that municipalities will collaborate with traditional leaders on matters affecting rural communities.

Ugu District Municipality adopted its Disaster Management By-law and published them on Government Gazette Number 1684 on 10 June 2016. This was a year after the adoption of the SFDRR and an opportune time for the alignment of the by-laws with the SFDRR priorities. With an interest in establishing consistency of the local-level regulatory framework and global frameworks, an enquiry underpinned in the cooperation theory was initiated. According to Catturani and Sacchetti (2017:6), cooperation theory applies in ‘symmetric structures, where actors occupy mutually dependent positions matched by expectations of reciprocity amongst peers’. In contextual terms, the relationship between traditional leaders and municipalities as *organs of state* should be guided by the cooperative governance principles prescribed in the *Constitution*. The guiding framework in relation to programmes and projects affecting traditional communities is the *cooperative rural governance model* envisaged in the *White Paper on Local Government* (Knoetze 2014:173). The basis for this cooperation is the fact that they are both legally empowered to serve the same communities and hence the requirement for their co-existence and the need for them to act in good faith and mutually respect the status for one another. The concept of *cooperative rural governance* recognises that both municipalities and traditional institutions have distinct but interrelated and interdependent functions that must be guided by cooperation protocols to avoid overlap and role conflict and, more importantly, reject competition.

One useful way of thinking through the provisions of the Disaster Management By-laws in relation to the participation of the traditional leadership is through Arnstein’s (1969) classical model of participation. Arnstein proposes eight rungs in ladder where the lowest rung entails manipulation and the top rungs represent full control by participants. Recently, there has been a renewed interest in the use of Arnstein’s (1969) classical model by scholars (cf. Ianniello et al. 2018; Simonsen 2018). On the contrary, Ansell and Gash (2008) emphasise the role of government in sectoral coordination towards collaborative governance. The interface of Arnstein’s model and Ansell and Gash’s theoretical perspective suggests a leading role of government, while participants like the traditional leadership take full advantage of the opportunity to influence processes.

### The human element in disaster risks

There is a degree of uncertainty around the definition of disaster among practitioners and scholars. A disaster, according to Twigg (2015:2), is an overwhelming destructive event to human, material, economy or environment triggered by natural or technological or human-made hazard or a combination of thereof, which exceeds the society’s ability to cope with. Twigg (2015:5) also states that ‘disaster events can sometimes set back years of economic and social development gains, generate political instability and cause long-lasting environmental damage.’ A disaster occurs when a hazard affects society or community. Twigg’s conceptualisation of a disaster is barely distinguishable from Mayner and Arbon’s (2015) characterisation, which also considers the destruction and disruption to a community as key elements to a disaster. Mayner and Arbon (2015) extend Twigg’s definition by offering more precise criteria for classifying an event like a disaster. This criterion considers the number of factors such as the number of people who die and/or affected and the response from the local and national government. This definition is useful in terms of precision, but it ignores the social, political and economic factors that may determine how the government responds to perceived disasters. The 2018 ‘Day Zero’ Western Cape South Africa provides a clear illustration of the complexity and limitations of clear-cut definitions of disaster. However, Mayner and Arbon’s (2015) conceptualisation of disaster is useful as it allows for an objective way of establishing what constitutes a disaster.

Another term that is often used interchangeably with disaster is a hazard. A hazard is:

[*A*] dangerous phenomenon, substance, human activity or condition that may cause loss of life, injury or other health impacts, property damage, loss of livelihoods and services, social and economic disruption or environmental damage. (Twigg 2015:25)

Drawing from electrical engineering, Arghandeh et al. (2016) provide a simple, yet incisive, definition of hazard. They describe it as an event or a set of events that have the potential to cause damage. These hazards can be classified into ‘natural (floods, earthquakes, landslides, windstorms), technological (industrial and transportation accidents) or those otherwise created by humans (riots, terrorist incidents and conflict)’ (Twigg 2015:2). The damage and loss caused by hazards is a product of vulnerability of the community or the society, which leads to disasters when hazards struck.

Twigg (2015:3) views vulnerability as the interplay between ‘economic, social, cultural, institutional and political factors’, which shape people’s lives and influence their environments. Twigg’s conceptualisation of vulnerability as function of natural and human factors is useful for this article as it allows one to think through how the Ugu District Municipality Disaster Management By-law on disaster risk management seeks to harness the social, political and economic factors and permit the active participation of local traditional leadership in managing disasters in the district. Disaster risk reduction, as Twigg points out, entails the ‘development and application of policies, strategies and practices to reduce vulnerabilities and disaster risks throughout society’. Drawing on UN Office for Disaster Risk Reduction (UNISDR), Twigg (2015) argues that disaster risk reduction concept and practice entails identifying and dealing with factors inducing disasters and remedying the exposure that reduces the vulnerability of people and property. These measures can include land and environmental management coupled with readiness in the event of a hazard striking.

Developments in the field of disaster management have led to a renewed interest in the nexus between people and the ecosystems. The interdependence between people and the ecosystem indicates that people’s behaviour can result in serious negative effects on it, thus affecting climate change (Twigg 2015). Twigg highlights a number of common mistakes that expose communities to flooding hazard, such as deforestation and erosion of vegetation, which happens during uncontrolled development; excessive grazing and cultivation weakens the soil and makes communities more susceptible to disaster risks. He posits that drought chances are more imminent if the vegetation is eroded as it fails the soil in its ability to hold water and allows it to run away and when weather releases the rain, rainwater cannot be absorbed to the soils and it flows over causing soil instability and flooding.

To Twigg (2015:20), one solution to all these states and challenges is the participatory management of the ecosystem that has an effect on climate change, implying a change of behaviour when people interact with it. Traditional leaders have a significant role to play in the changing of attitudes and transforming traditional practices such that they contribute to disaster risk reduction initiatives. Most traditional leaders in KwaZulu-Natal (KZN) exercise their authority in areas that fall under the Ingonyama Trust, and this land is estimated to be 30% of KZN land cover (Driver et al. 2015). Their role in relation to land allocation and usage is equally important to the customs and traditions that influence the customary way of life in traditional communities. Despite traditional leaders having authority to disallow certain practices that may expose communities to hazards, and also structured to enforce adopted rules, they equally need to fully understand the implications when they allocate land for various uses.

### Traditional institutions as a reinforcement of disaster risk reduction capacity

While the wave of urbanisation and westernisation is pervasive throughout South Africa and Africa at large, traditional leadership practices are enduring and unlikely to disappear overnight (Pearce 2018). However, it is not clear why traditional leadership seems to be taken for granted by policymakers and scholars alike. One possible explanation for this is that traditional leadership theory, which views power as centralised in one individual (Ferkins, Shilbury & O’Boyle 2017), has lost intellectual appeal because people have now embraced modern forms of governance. Consequently, there is a deafening dearth of literature on traditional leadership not only in disaster risk management, but also in governance in general. This leads to the underutilisation of human resources and lack of capacity. Capacity and resource constraints prove to be a challenge for effective disaster risk reduction initiatives. This claim is confirmed by Wentink and Van Niekerk (2017) who revealed damning empirical findings, indicating that municipalities are non-compliant with the minimum requirements for the establishing and operating Disaster Management Centres in relation to personnel, resources, equipment and disaster management structures. Twigg (2015) points out that there is no way that a sole institution can cope and be successful with disaster risk reduction because it requires a sophisticated and a multitude of knowledge, skills, attributes methods and resources. Sudden flood disasters may also be a challenge for disaster response given that there could be blockage of routes leading to the stricken communities should bridges and roads were flooded and washed away similarly to 1997, 2008 and 2011 flooding disasters within Ugu district (Carstens 2011).

One way in which traditional leadership can bolster the disaster management capacity is using centuries-old traditional mechanisms of managing disasters. Regarding this, Twigg (2015) and Ringo et al. (2016) reveal overwhelming indigenous coping mechanisms, which can be considered for integration to flood disaster risk reduction initiatives. In relation to personnel capacity, Luthuli (2015:28) reveals an institutional arrangement that can be very useful given that traditional leaders (AmaKhosi and Izinduna) are already on the government payroll as Public Office Bearers. According to Luthuli (2015:28), the traditional institution is formed in a hierarchical order from ‘iSilo, amaKhosi, headmen or headwomen, traditional councillors, traditional police officers’. This institution enjoys presence beyond the ward level and extends to Isigodi and sub-areas of Izigozi. It is obvious that the human resource element, which Wentink and Van Niekerk (2017) found to be crippling in municipalities, can be well augmented by tapping into this existing capacity.

In addition, 40% of the traditional council members is democratically elected by the community members to join the 60% that is appointed by Inkosi. This structure is assigned developmental functions (Knoetze 2014:180; Mashau, Mutshaeni & Kone 2014:221). These members together with the other non-paid members of the hierarchy can be best utilised as ‘volunteers’ because they are already in existence and recognised in the community. The other members of the society, normally known as ‘volunteers’, can be drawn in as additional capacity. All these people fall under the command of Inkosi and his Izinduna, and may easily become the champions of disaster risk reduction initiatives. They live and stay in the villages, making them the best first point of contact and resource for prompt response and recovery in the unfortunate event of disaster incidences. Moreover, they can serve as the best communication link with the communities and other groupings within the society that are expected to participate in disaster risk reduction initiatives.

## Methods

The study was underpinned by three overarching objectives:

- To ascertain articles of the Ugu District Municipality Disaster Management By-laws accommodating Cooperative Rural Governance in Disaster Risk Reduction Initiatives.
- To identify the strengths and weaknesses of the articles of the Ugu District Municipality Disaster Management By-laws accommodating Cooperative Rural Governance in Disaster Risk Reduction Initiatives.
- To recommend measures to strengthen the Ugu District Municipality Disaster Management By-laws with regard to Cooperative Rural Governance in Disaster Risk Reduction Initiatives.

To answer these questions, the study draws on the Ugu District Municipality Disaster Management By-laws. This required an immense engagement with the contents of the by-laws to establish the meaning and implications on cooperative rural governance. Ugu, with 39 traditional authorities, was seen as the perfect candidate for this case study. The study employed a non-obstructive method of ‘documents’. According to Auriacombe (2016:7), this method uses data from a wide range of written documents, including policy documents. This study targeted the policy document for Ugu District Municipality, which was accessed through the Internet-free platform from http://ugu.gov.za/Pages/Downloads.aspx.

A qualitative content analysis was used to extract words and sentences that relate to the role of traditional leaders from the by-laws. According to Bezuidenhout and Cronje (2014:234), qualitative content analysis, also known as textual analysis, is used to ‘explore and identify overt and covert themes and patterns embedded in a particular text’. The enumeration of sentences and keywords was based on the role or potential role for traditional leaders based on the focus of each chapter in the by-laws. The qualitative content analysis method was used. Content analysis, according to Bryman (2004; cited in Kohlbacher 2006:12), is ‘an approach to documents that emphasizes the role of the investigator in the construction of the meaning of texts’. Words and sentences contained in the by-laws were analysed in line with the research questions.

## Ethical considerations

This study meets the ethical standard set by the University of KwaZulu-Natal.

## Results and discussion

### Accommodation of traditional leadership in disaster risk reduction initiatives

This section presents an elaboration on findings from the review of the Ugu District Municipality’s Disaster Management By-laws. These by-laws are contained in Municipal Notice Number 69 of 2016 published in the Provincial Gazette Number 1684 of 10 June 2016. It encapsulates 11 chapters.

The first section of the *by-laws* is the Preamble to the By-laws and its Chapter 1 contains *Definitions*. Chapter 2 of the by-laws contain a reflection of the *status quo* pertaining to the Disaster Risk Profile of the District that influences disaster risk reduction initiatives and disaster risk management planning. This chapter also indicates the total number of traditional authorities and local municipalities that fall within the district. Interestingly, risk mitigation measures are also depicted against each disaster risk area. By implication, though not specifically stated, communities know how to deal with each of the identified risks.

The gist of the *cooperative rural governance model* should be reflected in Chapter 3, which encapsulates how community awareness initiatives should be coordinated within the district. Chapter 4 deals with *Public Safety* and it is where the issue of *saving lives* is articulated including the requirements and procedures for events. Chapter 5 reflects on measures to deal with disasters when they happen, including post-disaster initiatives for recovery and development. Chapter 6 informs about the operation of the *Disaster Management Centre* and its staffing. Chapter 7 defines the roles and responsibilities, while Chapter 8 reflects on the enforcement of the *by-laws.* This chapter indicates the circumstances that may lead to the declaration of a disaster and it prescribes procedural matters thereof. Chapter 9 is the general chapter followed by Chapter 10 that contains offences and related penalties enforceable, and Chapter 11, which is the last chapter, is the statement on the title of the *by-laws*.

Chapter 2 of the by-laws, as already indicated, reflects on the disaster risk profile. This chapter reflects a good risk profile per local municipality, although it does not zoom to specific traditional communities within each local municipality. The process that was followed to conduct the risk assessment is not discussed in this section. Under the circumstances, it is impossible to establish whether or not the process that was followed recognised the *cooperative rural governance* principles or rather the familiarity of traditional leaders with this profile. Chapter 3 deals with *Community Awareness* and its focus is on disaster risk management through the prioritisation of the transfer of necessary knowledge and skills to the community to prevent or minimise risks. Traditional leaders, however, do not feature on this chapter, although Article 3.1.a refers to *stakeholders and role players* and Article 3.1.b refers to *local community leaders*. These terms have not been covered under the *definitions section* and, as a result, it can only be assumed that *traditional leaders* are also inferred. The lack of specific reference to traditional leaders in these articles, however, opens chances for subjectivity and varying interpretations. Furthermore, an opportunity for mistaking the authority of traditional leaders in their communities is leveraged. The status and authority of traditional leaders may collapse under the suitcase words accommodating other stakeholders, role players and community leaders for various groupings within the community.

Article 3.2.b stipulates a need to include *indigenous knowledge* in disaster training programmes, although it is not indicated how such knowledge is to be obtained. While *indigenous knowledge* can also be obtained from non-rural areas, Ugu District Municipality serves more than 80% of the rural population that is led by traditional leaders who are custodians of customs and traditions upon which the generation-to-generation knowledge is preserved (Twigg 2015:139). In relation to disaster management, traditional leaders are legally required and expected to promote indigenous knowledge (Luthuli 2015:19). The role of traditional leaders in the preservation and validation of indigenous knowledge, which can be incorporated in the disaster management training material, is crucial. Article 3.3 uses the words *organ of state and other institutions*, and it is assumed that traditional leaders are included in the institutions referred to. However, the failure to, specifically refer to, traditional leaders makes their positions and role somewhat blur.

The evacuation of community members in cases of emergencies necessitating it is dealt with from Article 4.1 up to 4.6. However, there is no indication of how such evacuation will be coordinated in traditional communities in the absence of clauses dealing with traditional community-specific measures. This situation leaves it upon the discretion of those involved in the process, and there are great chances that the authority of an Inkosi of the affected area who has interest in the well-being of his people is overlooked or undermined. A point of entry in a traditional community ideally should be through its traditional leader given that he is vested with a supreme office of authority in that community (Luthuli 2015:32).

It must be noted that when people are evacuated, other things like livestock and belongings might be left behind and Inkosi of the area together with Izinduna and the entire traditional community system must play a vigilant role to prevent intruders coming and taking advantage of the victims of the disaster. Similarly, should livestock and domestic animals be dead as a result of the disaster, the traditional leadership system plays a significant role to manage their disposal processes. Those people who are evacuated should be able to return to the community and continue with the social life of the community after the emergency. Article 4.7 deals with events including approval for such events. The role of Inkosi where the event is to take place is not defined. There is a possibility that the municipality may approve everything relating to the event, but only to find that the event itself did not get the approval in terms of the customs and traditions of the community. Should such a situation prevail, it is clear that the Inkosi’s authority over his community would have been compromised. Unfortunately, this situation is contrary to the South African legislative environment and the vision of the SFDRR.

Article 5.1.1 uses the word *authorities* and it can only be assumed that it also refers to Inkosi. The composition of the Disaster Management Advisory Forum does not include AmaKhosi. The only term that is closely related is the *relevant community-based organisations*, which appears on the list of possible members. It is not clear whether or not this term is meant to include AmaKhosi. It can also be argued that categorising AmaKhosi under *community-based organisation* may be inappropriate as the term puts them under one basket with various interest groups that may have formed within the community. In this regard, the by-laws do not explicitly cater for traditional leaders to serve in the Ugu District Disaster Management Forum and yet its area is more than 80% rural. Not much relates to the role of traditional leaders in relation to Article 6 that deals with the *Disaster Management Centre*, although a weakness is identified in relation to communication system and methods that are silent about traditional leaders. Traditional leaders, though, can serve as a reliable system to communicate to traditional communities based on its entrenched traditional modes of communication.

Article 7.7.2 refers to the voluntary participation of community members in the advisory forum. This statement is vague as it is impossible to have the entire community participating on a voluntary basis, and this clause raises a question as to how this voluntary participation is supposed to be managed. Article 8 deals with the declaration of disasters and despite this section touching communities directly, there is no role for traditional leaders reflected; only elected public representatives have been accommodated in relation to all processes concerning the declaration of disasters and subsequent activities. The rest of the chapters have no issues in relation to traditional leaders.

The study revealed that Ugu District Municipality Disaster Management By-laws vaguely accommodate the cooperative model for rural governance. Traditional leaders are not even listed in Article 7.2, which lists stakeholders who may become members of the Municipal Disaster Advisory Forum. Reference to traditional leaders is only made in Article 7.8 in a 43-page By-laws. It also appears that this reference is meant for those traditional leaders that are participating in Ugu District Municipality in terms of Section 81 of the *Municipal Structures Act* through the reference to Article 7.6 dealing with Responsibilities of the Ugu District Municipality Council.

These by-laws, however, make recognition of the fact that the role of the community in disaster management initiatives is vital. It further attempts to indicate that there are stakeholders who have a major role to play in disaster governance matters. However, these by-laws are very weak in pronouncing specifically to traditional leaders *per se* and yet the district is more than 80% rural. However, Ugu District Municipality Disaster Management By-laws in isolation might lead to serious flaws in terms of how we understand the disaster governance in South Africa. One needs to appreciate that the by-laws do work independently of other policies and legislation that regulate local government governance. For instance, disaster management is a key component of all municipal Integrated Development Plans (IDPs), which engender strong public participation. Reading the by-laws isolation will be to ignore other constitutional and national legislative frameworks that regulate the activities of municipalities.

Nevertheless, the lack of emphasis on the traditional leadership in disaster management might reflect Arnstein’s (1969) lower level rungs, which emphasise manipulation, therapy and informing without true-delegated power, partnership and control. While these by-laws may not be the best in terms of accommodating traditional leadership, they are better than some municipal by-laws in that regard. For instance, a five-chapter city of Umhlathuze Disaster Management By-laws does not mention the traditional leadership at all. Again, it may not mean that such traditional authorities are not participating in disaster management. However, if the roles of traditional authorities are not clearly stipulated in the by-laws, one can argue that such participation could be merely a tokenism gesture (Ianniello et al. 2018; Simonsen 2018). The use of by-laws to regulate municipal activities is crucial as it seeks to be relevant towards local needs of a local government, which might not be specified or adequately catered for by provincial or national legislation given the diversity of communities served at the local level.

### Measures to strengthen the Ugu District Municipality Disaster Management By-laws

The findings reveal that the cooperative rural governance model has been loosely accommodated in the Ugu District Municipality Disaster Management By-laws. Traditional leaders could be specifically referred to under Section 3 dealing with ‘community awareness’ to limit and avoid any possible discretional interpretation. Where indigenous knowledge is to be obtained from traditional leaders, this can be done in an orderly manner and this could include presentations done to traditional leaders on training material at its development stage. The custodians of indigenous knowledge are traditional leaders and they must be accorded recognition as such so as to legitimise any knowledge purported to be indigenous.

The evacuation of rural people needs to be done in collaboration with the Inkosi of the area concerned together with his governance system. The process flow for the coordination and regulation of events taking place within the traditional leader’s area could include Inkosi throughout until the venue is ultimately cleared off and left in its original state as it was before the event. Traditional leaders, in cognisance of the cooperative model for rural governance, must be listed on the composition for the *Disaster Management Forum*. Section 17(3) of the *Traditional Leadership and Governance Framework Act* provides for the role of the *Local House of Traditional Leaders* and this role is for providing advice to the District Municipality. It is abnormal that traditional leaders are not specifically listed on a district structure for disaster management coordination in a predominantly rural municipality. If there is a desire to have community representatives in the Disaster Management Advisory Forum, such could be clearly articulated, and it must also be clear how such representation should take place. However, such representation should not replace traditional leadership representation. Replacing traditional leaders with community members in structures of this nature can be construed as a derogation of the constitutional authority of traditional leaders to their communities.

Article 6 needs to be strengthened to include traditional institutions serving as one best-fit method to send information to traditional communities. This is an institution that governs through traditional systems and these systems are better understood than any other new or modern systems that are foreign to their customary way of life. There is a likelihood of failing to reach communities if such traditional modes of communication are overlooked. As Luthuli (2015:42) argues, this is an institution that already exists and structured right from the neighbourhood where traditional police and Izinduna are found. These are people who live and stay in these communities and will always be there whenever there is a need.

Article 7.8 specifies that AmaKhosi and Councillors are required to comply with the by-laws, and such a statement is vague. The context within which the specified compliance is desired could be made clear. Article 8 dealing with the declaration of disasters needs to be strengthened to reflect a clear role of traditional leaders as it is impossible that government representatives can get into the area ruled by Inkosi and just give orders. The Inkosi and his traditional council are the ones who accept people into their community (Alcock & Hornby 2004:11), and they are better positioned to know conditions in each and every household within the society. They are too close to the community and, by default, they will be the ones to arrive on disaster scenes even before the government authorities do. Inadequate policies create implementation challenges when practice meets reality on the ground. It is in this regard that the local government is also required to formulate clearly articulated and enabling policies to avoid interpretation challenges.

## Conclusion

While modern forms of governance, which shun traditional forms of leadership, have an intuitive appeal among the urbanised communities and scholars, its application in rural areas such Ugu, which is over 80% rural, may not be appropriate and effective. This article has illustrated that Disaster Management By-laws on disaster risk management that does not explicitly state the role of traditional leadership fall short in terms of Cooperative Rural Governance in Ugu district. Such by-laws tend to cater for a small proportion of people who do not live under the jurisdiction of traditional leaders. In view of the Sendai Framework on Disaster Risk Reduction, this does not seem to empower local authorities and communities as envisioned by the framework. While providing partial answers, this study requires a new set of questions asked. The relevance of urban sensitive by-laws to rural communities that are governed by traditional leaders needs to be interrogated further, particularly in view of disaster management. Ways in which municipalities and traditional institutions can be harmonised through clear by-laws to enhance the cooperative model for rural governance should be sought and pursued. However, caution must be exercised when analysing local municipality by-laws. One should not read them in isolation and independent of the provincial and national legislation. While the findings of this study are of interest to local government officers, they also provide a springboard for further research and in the field of disaster risk management. More precise questions at a larger scale can be asked to elicit insights into the regulative framework for traditional leadership to participate in disaster risk management.
